# Nivolumab plus ipilimumab-based treatment in Japanese patients with metastatic non-small cell lung cancer and tumor PD-L1 < 1%: a pooled analysis

**DOI:** 10.1007/s10147-026-03116-w

**Published:** 2026-08-11

**Authors:** Makoto Nishio, Hidetoshi Hayashi, Yasushi Goto, Satoshi Ikeda, Toshihide Yokoyama, Hiroshi Tanaka, Yuichi Tambo, Katsuyuki Hotta, Tatsuro Fukuhara, Haruko Daga, Ken Masubuchi, Takekazu Aoyama, Marina Tschaika, Nan Hu, Hideaki Mizutani

**Affiliations:** 1 Thoracic Medical Oncology Department, Cancer Institute Hospital of the Japanese Foundation for Cancer Research, 3-8-31, Ariake, Koto, Tokyo 135-8550 Japan; 2 Department of Medical Oncology, Faculty of Medicine, Kindai University, Osaka, Japan; 3 Department of Thoracic Oncology, National Cancer Center Hospital, Tokyo, Japan; 4 Department of Respiratory Medicine, Kanagawa Cardiovascular and Respiratory Center, Yokohama, Kanagawa Japan; 5 Department of Respiratory Medicine, Kurashiki Central Hospital, Kurashiki, Okayama Japan; 6 Department of Internal Medicine, Niigata Cancer Center Hospital, Chuo Ward, Niigata, Japan; 7 Department of Respiratory Medicine, Kanazawa University Hospital, Kanazawa, Ishikawa Japan; 8 Department of Respiratory Medicine, Okayama University Hospital, Kita Ward, Okayama, Japan; 9 Tatsuro Fukuhara, Miyagi Cancer Center, Natori, Miyagi Japan; 10 Department of Medical Oncology, Osaka City General Hospital, Chuo Ward, Osaka, Japan; 11 Division of Respiratory Medicine, Gunma Prefectural Cancer Center, Ota, Gunma Japan; 12 Bristol Myers Squibb Company, Princeton, NJ USA; 13 Department of Thoracic Oncology, Saitama Cancer Center, Kitaadachi District, Saitama, Japan

**Keywords:** Japan, Immunotherapy, Nivolumab, Ipilimumab, Tumor PD-L1, Non-small cell lung cancer

## Abstract

**Background:**

Nivolumab plus ipilimumab-based therapies have shown long-term, durable clinical benefit in patients with metastatic non-small cell lung cancer (NSCLC), including those with tumor programmed death-ligand 1 (PD-L1) expression < 1%, a population with high unmet need. Here we report long-term clinical outcomes with first-line nivolumab plus ipilimumab with or without chemotherapy (2 cycles) versus chemotherapy (≤ 4 cycles) in a pooled population of Japanese patients with metastatic NSCLC and tumor PD-L1 < 1% from the randomized, phase 3 CheckMate 227 (NCT02477826) and CheckMate 9LA (NCT03215706) studies.

**Methods:**

Adults with stage IV/recurrent NSCLC without sensitizing *EGFR*/*ALK* alterations were included. Overall survival (OS), progression-free survival (PFS), objective response rate, duration of response, and safety were assessed.

**Results:**

Among the pooled population of Japanese patients with tumor PD-L1 < 1% in the nivolumab plus ipilimumab with or without chemotherapy (*n* = 34) versus chemotherapy (*n* = 41) arms, median OS was 41.0 (95% CI 19.4–not reached) versus 15.2 (95% CI 7.6–21.3) months (hazard ratio [HR] 0.46; 95% CI 0.27–0.78); 5-year OS rates were 38% (95% CI 22–54) versus 16% (95% CI 6–30). Median PFS was 8.2 (95% CI 4.1–19.3) versus 5.6 (95% CI 4.2–7.0) months (HR 0.57 [95% CI 0.31–1.03]). No new safety signals were observed.

**Conclusions:**

Consistent with results in the pooled global population, nivolumab plus ipilimumab with or without chemotherapy showed long-term, durable clinical benefit in Japanese patients with metastatic NSCLC and tumor PD-L1 < 1%, suggesting potential clinical benefit with its use as a first-line treatment for this hard-to-treat population.

**Supplementary Information:**

The online version contains supplementary material available at https://doi.org/10.1007/s10147-026-03116-w.

## Introduction

Programmed death 1 (PD-1)/programmed death ligand 1 (PD-L1) inhibitor-based immunotherapy has significantly improved long-term survival outcomes versus chemotherapy alone in the first-line setting in patients with metastatic non-small cell lung cancer (NSCLC) [1–7]. Tumor PD-L1 expression level is widely used as a predictive biomarker of response to anti-PD-1/PD-L1-based immunotherapy in metastatic NSCLC, with evidence indicating patients with high versus low tumor PD-L1 expression were more likely to derive treatment benefit from anti-PD-1/PD-L1 monotherapy [8–11]. Several PD-1/PD-L1 inhibitors, such as pembrolizumab, atezolizumab, and cemiplimab, are currently approved as first-line treatment for patients with advanced NSCLC and high tumor PD-L1 expression [12–14]. However, patients with tumor PD-L1 < 1% tend to experience limited efficacy benefit with these therapies, indicating a high unmet medical need for this patient population [4, 5, 7, 15].

Nivolumab, a fully human PD-1 antibody, and ipilimumab, a fully human cytotoxic T-lymphocyte antigen 4 antibody, have distinct but complementary mechanisms of action [16–18]. Data from two pivotal randomized, phase 3 studies, CheckMate 227 (NCT02477826) Part 1 and CheckMate 9LA (NCT03215706), showed significant overall survival (OS) benefit with first-line nivolumab plus ipilimumab with or without chemotherapy versus chemotherapy alone in patients with metastatic NSCLC, including those with tumor PD-L1 < 1% [19, 20]. On the basis of these results, nivolumab plus ipilimumab is approved as first-line treatment in the United States and other countries for patients with metastatic NSCLC with no *EGFR*/*ALK* aberrations and tumor PD-L1 ≥ 1%, and in Japan as first-line treatment regardless of tumor PD-L1 expression [21–23]. Nivolumab plus ipilimumab with two cycles of platinum-doublet chemotherapy is approved as first-line treatment in several countries, including the United States, the European Union, and Japan, for patients with metastatic NSCLC with no *EGFR*/*ALK* aberrations, regardless of tumor PD-L1 expression [21–24].

Patients with tumor PD-L1 < 1% continued to derive long-term, durable clinical benefit with nivolumab plus ipilimumab with or without chemotherapy versus chemotherapy alone. At the 6-year follow-up of CheckMate 227 Part 1 in patients with tumor PD-L1 < 1%, the hazard ratio (HR) for OS with nivolumab plus ipilimumab versus chemotherapy was 0.65 (95% confidence interval [CI] 0.52–0.81) and 6-year OS rates were 16% versus 5%; 25% versus 0% of responders had ongoing responses at 6 years [1]. At the final 6-year update of CheckMate 9LA, patients with tumor PD-L1 < 1% had an OS HR of 0.64 (95% CI 0.49–0.84) with nivolumab plus ipilimumab with chemotherapy versus chemotherapy, with 6-year OS rates of 20% versus 7%; 25% versus 0% of responders had ongoing responses at 6 years [2]. Long-term, durable clinical benefit with nivolumab plus ipilimumab with or without chemotherapy was also observed in a large, global, pooled analysis of patients from CheckMate 227 and CheckMate 9LA with tumor PD-L1 < 1% [25]. At 5-year follow-up, median OS was 17.4 months with nivolumab plus ipilimumab with or without chemotherapy versus 11.3 months with chemotherapy (HR 0.64 [95% CI 0.54–0.76]), and 5-year OS rates were 20% versus 7%; the median duration of response (DOR) was 18.0 versus 4.6 months, with one-fourth of responders experiencing ongoing responses at 5 years [25].

Differences in treatment outcomes between Asian and non-Asian populations with NSCLC have been reported, possibly owing to factors such as inherent genetic variations, differences in healthcare systems, and epidemiological and demographic differences, which could impact disease course [26]. It is, therefore, important to evaluate clinical outcomes in Japanese patients to better optimize treatment strategies for this subpopulation.

Both CheckMate 227 and CheckMate 9LA evaluated first-line treatment strategies based on the dual immune-checkpoint blockade of nivolumab plus ipilimumab, with or without a limited course of chemotherapy. Given the shared therapeutic backbone between the two studies and the limited long-term data available for Japanese patients with tumor PD-L1 < 1%, we conducted pooled analyses to better characterize efficacy outcomes in this subpopulation. Here, we present long-term efficacy and safety outcomes of nivolumab plus ipilimumab with or without chemotherapy versus chemotherapy alone in a pooled population of Japanese patients with metastatic NSCLC and tumor PD-L1 < 1% from the CheckMate 227 and CheckMate 9LA studies.

## Patients and methods

### Patients

The global, randomized, open-label, phase 3 studies CheckMate 227 (NCT02477826) and CheckMate 9LA (NCT03215706) evaluated regimens based on the dual immunotherapy combination of nivolumab plus ipilimumab as first-line treatment for patients with metastatic NSCLC. The eligibility criteria for these two studies have been reported previously [19, 20, 27]. Briefly, enrolled patients were adults with histologically confirmed squamous or non-squamous stage IV or recurrent NSCLC, with no known *EGFR* mutations or *ALK* translocations, an Eastern Cooperative Oncology Group (ECOG) performance status of 0–1, and who had received no prior systemic anticancer therapy [19, 20]. This exploratory post hoc analysis only included Japanese patients with tumor PD-L1 < 1% from CheckMate 227 Part 1 and CheckMate 9LA.

CheckMate 227 and CheckMate 9LA were conducted in accordance with the Declaration of Helsinki and International Standards of Good Clinical Practice. The independent ethics committee or institutional review board of each participating study center approved the protocols and all amendments. All patients provided written informed consent.

### Study designs and treatment

The study designs for CheckMate 227 Part 1 and CheckMate 9LA have been described previously [19, 20, 27]. In CheckMate 227 Part 1, patients with tumor PD-L1 ≥ 1% were randomly assigned 1:1:1 to receive nivolumab (3 mg/kg every 2 weeks [Q2W]) plus ipilimumab (1 mg/kg every 6 weeks [Q6W]), nivolumab monotherapy (240 mg Q2W), or platinum-doublet chemotherapy (every 3 weeks [Q3W] for ≤ four cycles). Patients with tumor PD-L1 < 1% were randomly assigned 1:1:1 to receive nivolumab plus ipilimumab, nivolumab (360 mg Q3W) plus platinum-doublet chemotherapy (≤ four cycles Q3W), or platinum-doublet chemotherapy (Q3W for ≤ four cycles) [19]. Patients were stratified by tumor histology (squamous vs. non-squamous).

In CheckMate 9LA, patients were randomly assigned 1:1 to receive nivolumab (360 mg Q3W) plus ipilimumab (1 mg/kg Q6W) combined with platinum-doublet chemotherapy (Q3W for two cycles) or chemotherapy alone (Q3W for four cycles). Patients were stratified by tumor histology (squamous vs. non-squamous), sex (male vs. female), and tumor PD-L1 expression (< 1% vs. ≥ 1%). Optional pemetrexed maintenance (500 mg/m^2^) was permitted in patients with non-squamous histology in the chemotherapy arm [20].

In both studies, Japan as a geographical region was not a stratification factor. Treatment continued until disease progression, unacceptable toxicity, or for up to 2 years for immunotherapy.

### Endpoints and assessments

The primary endpoints for the individual CheckMate 227 (OS with nivolumab plus ipilimumab vs. chemotherapy in patients with tumor PD-L1 ≥ 1%) and CheckMate 9LA (OS with nivolumab plus ipilimumab with chemotherapy vs. chemotherapy) studies have been described previously [19, 20].

The efficacy outcomes in this exploratory post hoc analysis included OS (overall and in key patient subgroups), progression-free survival (PFS) per blinded independent central review (BICR), best overall response (BOR) per Response Evaluation Criteria in Solid Tumors (RECIST) v1.1, objective response rate (ORR) per RECIST v1.1, and DOR per BICR (defined as the time between date of first confirmed documented response and date of first documented tumor progression per RECIST v1.1, death, or last tumor assessment before subsequent therapy, whichever occurred first). Efficacy outcomes were also reported for patients who discontinued all components of nivolumab plus ipilimumab (with or without chemotherapy) due to treatment-related adverse events (TRAEs; defined as all AEs reported between first dose of study drug and 30 days after last dose). Safety was reported in patients who received at least one dose of study drug.

Tumor PD-L1 expression was determined using the PD-L1 IHC 28−8 pharmDx assay (Agilent Dako, Santa Clara, CA, USA), as described previously [28]. AEs were graded according to the National Cancer Institute Common Terminology Criteria for Adverse Events (CTCAE) v4.0 and Medical Dictionary for Regulatory Activities (MedDRA) v25.1 for CheckMate 227 and v26.1 for CheckMate 9LA.

### Statistical analyses

Survival curves and rates of OS, PFS, and DOR were estimated using the Kaplan–Meier methodology. HRs and associated 95% CIs were estimated using unstratified Cox proportional hazard models with treatment arm as a single covariate. CIs for ORR were based on the Clopper–Pearson method.

## Results

### Patients and treatment

In the pooled cohort of Japanese patients with tumor PD-L1 < 1%, 34 patients were included in the nivolumab plus ipilimumab with or without chemotherapy arm (*n* = 25 with nivolumab plus ipilimumab from CheckMate 227 and *n* = 9 with nivolumab plus ipilimumab with chemotherapy from CheckMate 9LA), and 41 patients were included in the chemotherapy arm (*n* = 29 from CheckMate 227 and *n* = 12 from CheckMate 9LA) (Supplementary Fig. 1). Baseline characteristics were generally balanced between the treatment arms (Table 1); however, a higher proportion of patients had brain metastases in the nivolumab plus ipilimumab with or without chemotherapy arm (12%) versus the chemotherapy arm (2%).

**Table 1:** Demographics and baseline characteristics in the pooled tumor PD-L1 < 1% Japanese subpopulation

*n* (%)	Nivolumab plus ipilimumab with or without chemotherapy (*n* = 34)	Chemotherapy (*n* = 41)
Age, median (range), years	67.5 (42–81)	67.0 (30–78)
< 65	15 (44)	17 (41)
65–75	15 (44)	20 (49)
≥ 75	4 (12)	4 (10)
Male	29 (85)	33 (80)
ECOG PS		
0	16 (47)	19 (46)
1	18 (53)	22 (54)
Smoking status		
Current/former	27 (79)	35 (85)
Never	7 (21)	6 (15)
Histology type		
Squamous	7 (21)	8 (20)
Non-squamous	27 (79)	33 (80)
Disease stage		
Stage IV	29 (85)	38 (93)
Recurrence	5 (15)	3 (7)
Metastases^a^		
Brain	4 (12)	1 (2)
Liver	5 (15)	5 (12)
Bone	8 (24)	7 (17)
Prior therapy^b^		
Platinum-based chemotherapy	1 (3)	1 (2)
Other chemotherapy	2 (6)	2 (5)

^a^Per BICR

^b^Included prior therapy for early-stage (i.e., not metastatic or recurrent) disease

*BICR* blinded independent central review; *ECOG PS* Eastern Cooperative Oncology Group performance status; *PD-L1* programmed death ligand 1

At database lock (CheckMate 227: 21 February 2023; CheckMate 9LA: 15 December 2023), the median follow-up for the pooled Japanese subpopulation was 76.1 (range 57.3–85.1) months. Median duration of treatment was 3.5 (range 0–24.3) months, with patients receiving a median of eight doses of nivolumab (range 1–53) and four doses of ipilimumab (range 1–18), which was similar to that reported for the pooled global population (Supplementary Table 1) [25].

Most patients in the pooled Japanese subpopulation received any subsequent anticancer therapy (nivolumab plus ipilimumab with or without chemotherapy, 82%; chemotherapy, 93%); 21% of patients in the nivolumab plus ipilimumab with or without chemotherapy arm and 71% of patients in the chemotherapy arm received subsequent immunotherapy (Supplementary Table 2). Of the six patients who did not receive any subsequent therapy in the nivolumab plus ipilimumab with or without chemotherapy arm, three were alive as of database lock and had sustained tumor response beyond two years after treatment initiation, although one patient later experienced disease progression. Of the remaining three patients, two had disease progression and died early and one died early with no documented progression (Supplementary Fig. 2). Among the patients in the nivolumab plus ipilimumab with or without chemotherapy arm who did not receive any subsequent therapy, baseline characteristics were generally similar between those with sustained tumor response and those who died before the database lock date; however, some differences in metastasis sites were observed between the two subgroups (Supplementary Fig. 2).

### Efficacy in the pooled Japanese subpopulation of patients with tumor PD-L1 < 1%

**Fig. 1:**
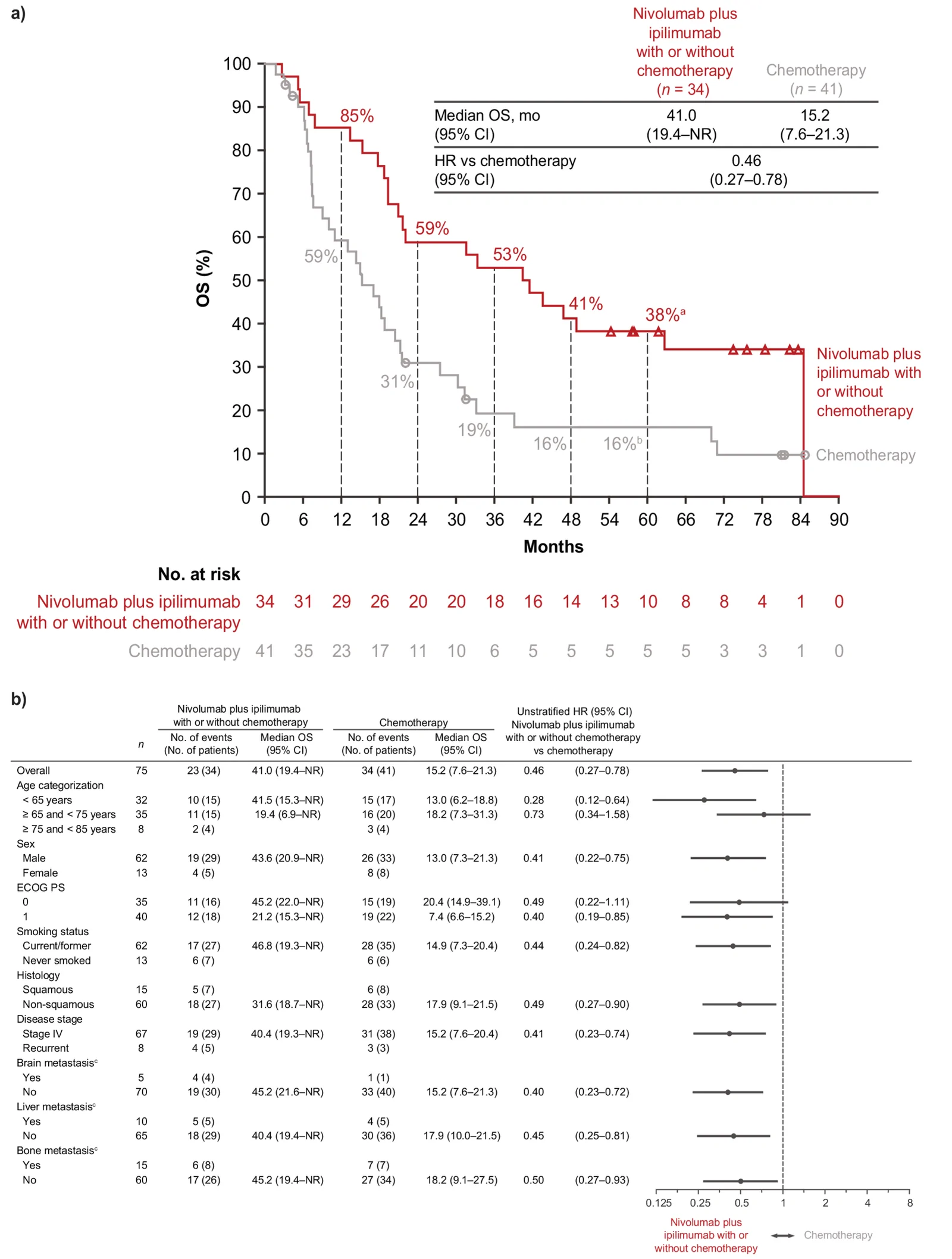
OS in the pooled Japanese subpopulation with tumor PD-L1 < 1% (**a)** in all patients and (**b)** by key patient subgroups. HR and median values were not computed for subgroup categories with < 10 events per treatment arm. ^a^95% CI, 22–54. ^b^95% CI, 6–30. ^c^Per BICR. *BICR* blinded independent central review; *Chemo* chemotherapy; *CI* confidence interval; *ECOG PS* Eastern Cooperative Oncology Group performance status; *HR* hazard ratio; *IPI* ipilimumab; *mo* months; *NIVO* nivolumab; *OS* overall survival; *PD-L1* programmed death ligand 1

In the pooled Japanese subpopulation with tumor PD-L1 < 1%, median OS was 41.0 (95% CI 19.4–not reached) months with nivolumab plus ipilimumab with or without chemotherapy versus 15.2 (95% CI 7.6–21.3) months with chemotherapy (hazard ratio [HR] 0.46; 95% CI 0.27–0.78); 5-year OS rates were 38% (95% CI 22%–54%) versus 16% (95% CI 6%–30%) (Fig. 1a). OS benefit was also observed across key patient subgroups, including those defined by age, sex, smoking status, tumor histology type, and ECOG performance status (Fig. 1b).

**Fig. 2:**
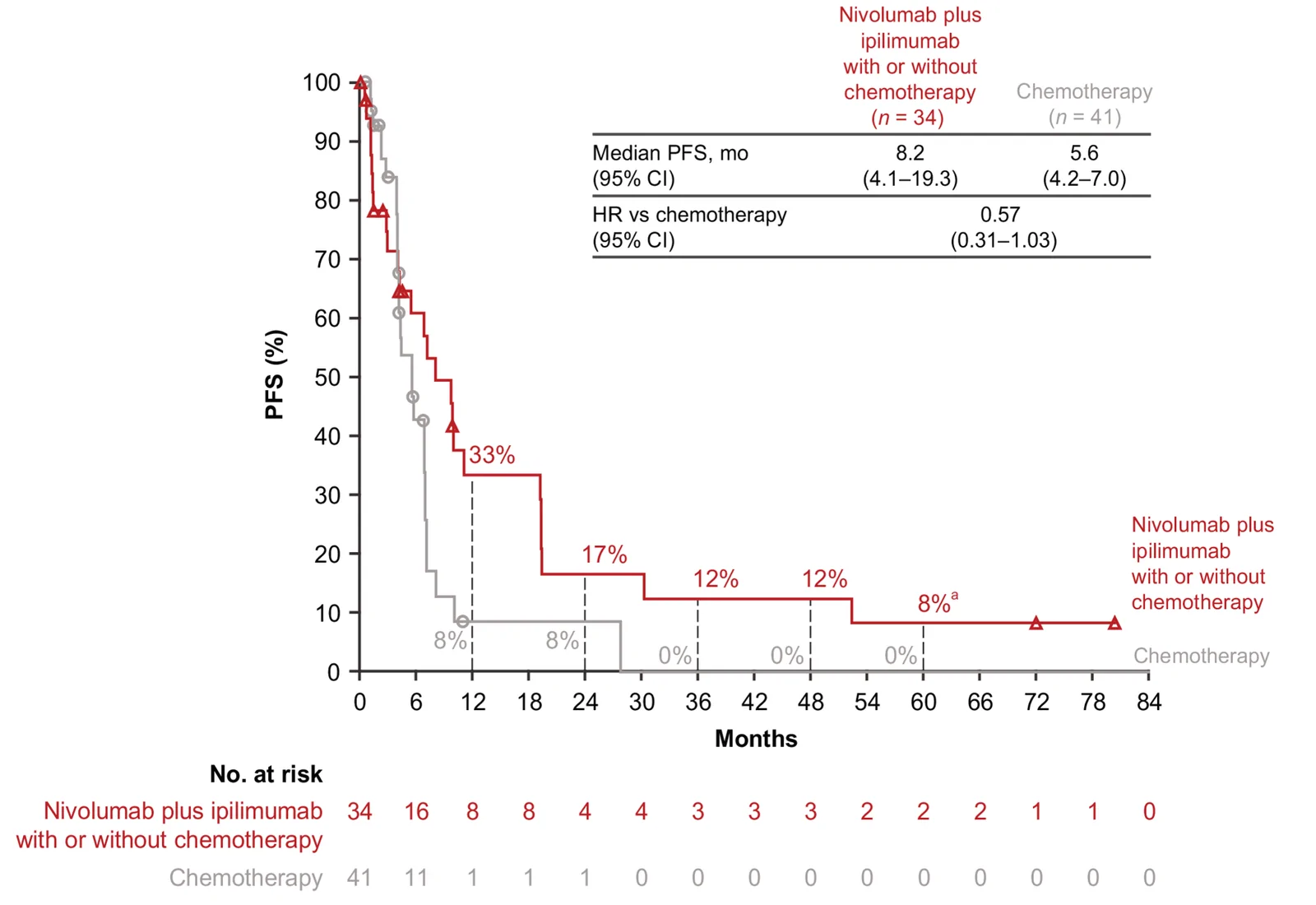
PFS per BICR in the pooled Japanese subpopulation with tumor PD-L1 < 1%. ^a^95% CI, 2–23. *BICR* blinded independent central review; *Chemo* chemotherapy; *CI* confidence interval; *HR* hazard ratio; *IPI* ipilimumab; *mo* months; *NIVO* nivolumab; *PD-L1* programmed death ligand 1; *PFS* progression-free survival

**Fig. 3:**
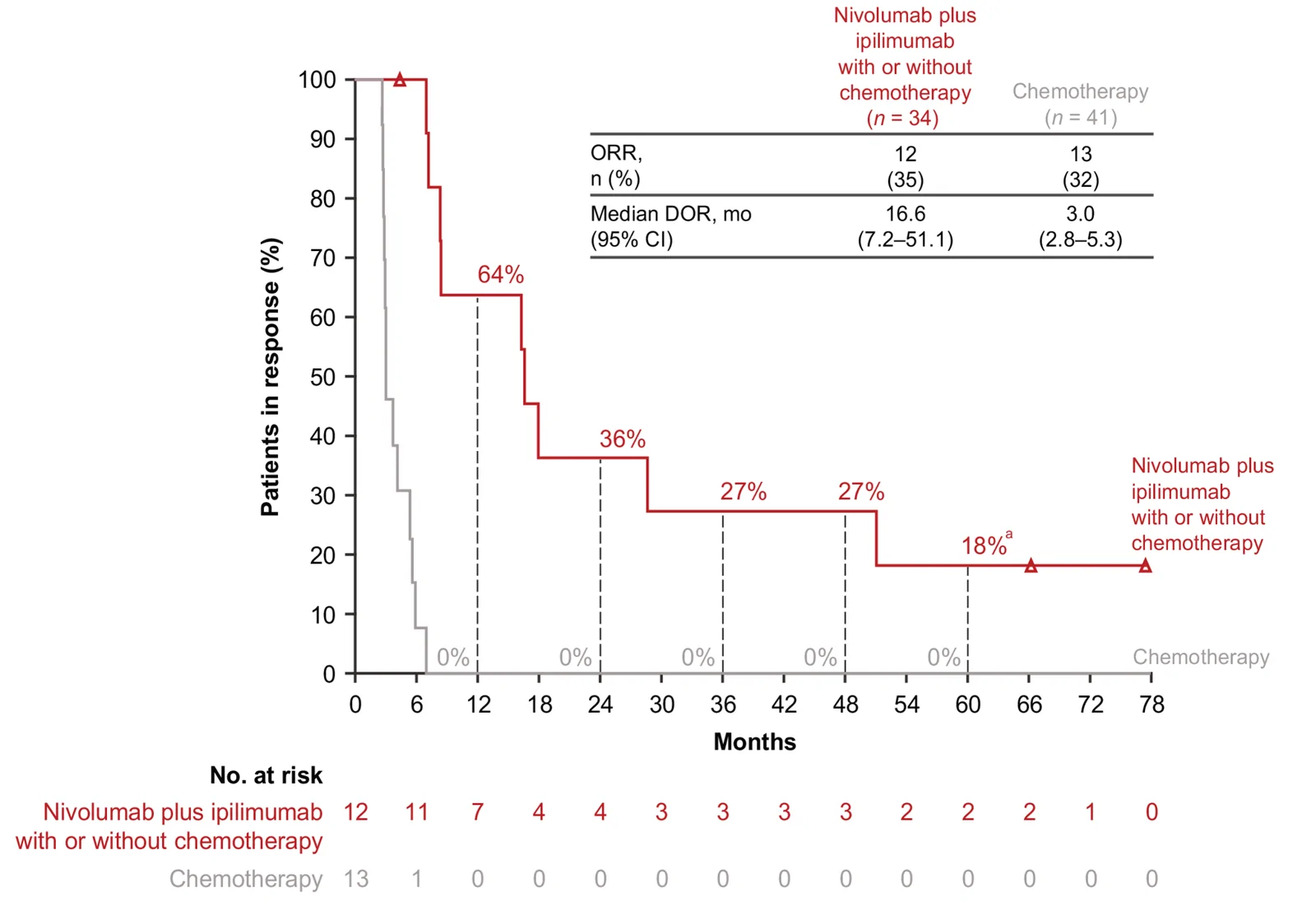
DOR per BICR in the pooled Japanese subpopulation with tumor PD-L1 < 1%. ^a^95% CI, 3–44. *BICR* blinded independent central review; *CI* confidence interval; *Chemo* chemotherapy; *DOR* duration of response; *HR* hazard ratio; *IPI* ipilimumab; *mo* months; *NIVO* nivolumab; *ORR* objective response rate; *PD-L1* programmed death ligand 1

Median PFS per BICR in the pooled population was 8.2 (95% CI 4.1–19.3) months with nivolumab plus ipilimumab with or without chemotherapy versus 5.6 (95% CI 4.2–7.0) months with chemotherapy alone (HR 0.57; 95% CI 0.31–1.03); 5-year PFS rates were 8% (95% CI 2–23) versus 0% (Fig. 2). The ORR was 35% (95% CI 20–54) with nivolumab plus ipilimumab with or without chemotherapy versus 32% (95% CI 18–48) with chemotherapy, and median DOR was 16.6 (95% CI 7.2–51.1) versus 3.0 (95% CI 2.8–5.3) months, with ongoing responses at 5 years observed in 18% (95% CI 3%–44%) versus 0% of responders (Fig. 3; Supplementary Table 3).

### Efficacy in the pooled Japanese subpopulation of patients with tumor PD-L1 < 1% and who discontinued treatment due to TRAEs

**Fig. 4:**
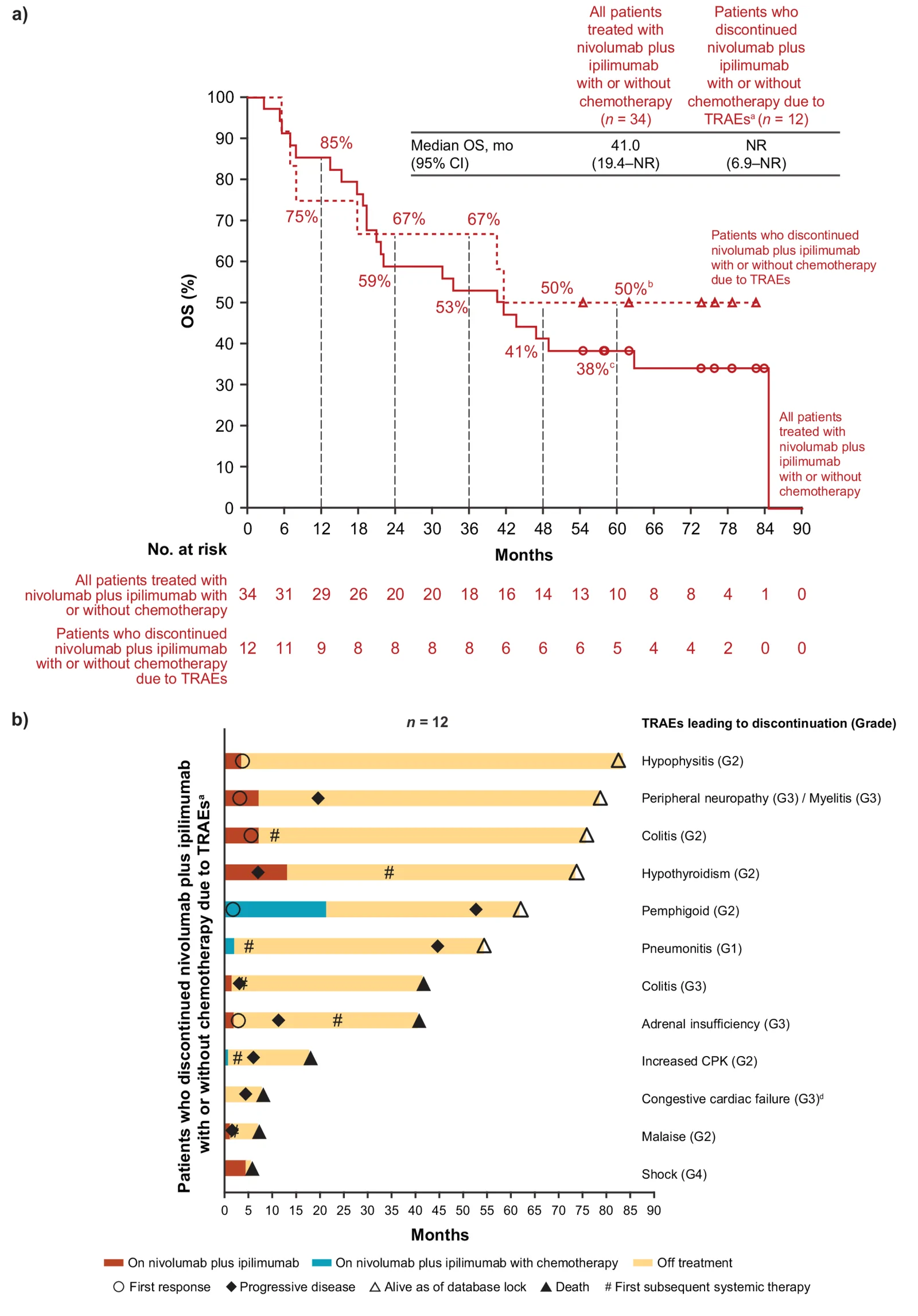
OS (**a**) and treatment status and response (**b**) in the pooled Japanese subpopulation with tumor PD-L1 < 1% who discontinued nivolumab plus ipilimumab with or without chemotherapy due to TRAEs. ^a^Patients discontinued all components of nivolumab plus ipilimumab with or without chemotherapy due to TRAEs. ^b^95% CI, 22–54. ^c^95% CI, 21–74. ^d^On nivolumab plus ipilimumab. *CI* confidence interval, *Chemo* chemotherapy; *CPK* creatine phosphokinase; *DBL* database lock; *Ipi* ipilimumab; *mo* months; *Nivo* nivolumab, *NR* not reached; *OS* overall survival; *PD-L1* programmed death ligand 1; *TRAE* treatment-related adverse event

Of the patients treated with nivolumab plus ipilimumab with or without chemotherapy, 12 (35%) discontinued all components of the treatment regimen due to TRAEs. The median OS was not reached, and the 5-year OS rate was 50% (6 of 12 patients) (Fig. 4a). At database lock, seven patients in this subgroup had received subsequent systemic therapy, of whom three were still alive. Duration of treatment and response patterns in Japanese patients who discontinued all components of treatment due to TRAEs are shown in Fig. 4b.

### Safety

Overall, no new safety signals were identified in the pooled Japanese subpopulation. The most common TRAEs of any grade (in ≥ 15% of patients) reported with nivolumab plus ipilimumab with or without chemotherapy were malaise (29%), maculo-papular rash (29%), decreased appetite (21%), fatigue (21%), constipation (18%), hyperthyroidism (18%), pyrexia (18%), increased alanine aminotransferase (15%), and rash (15%); the most common grade 3–4 TRAE (in ≥ 5% of patients) was maculo-papular rash (6%) (Table 2). In the chemotherapy arm, the most common TRAEs of any grade (in ≥ 15% of patients) were constipation (54%), nausea (51%), decreased appetite (37%), anemia (34%), hiccups (32%), decreased platelet count (32%), decreased neutrophil count (29%), malaise (24%), increased alanine aminotransferase (15%), and neutropenia (15%); the most common grade 3–4 TRAEs (in ≥ 5% of patients) were anemia (15%), decreased neutrophil count (15%), neutropenia (10%), decreased appetite (7%), decreased platelet count (7%), and decreased white blood cell count (7%) (Table 2). Serious TRAEs of any grade were reported in 38% of patients in the nivolumab plus ipilimumab with or without chemotherapy arm and 24% of patients in the chemotherapy arm; grade 3–4 serious TRAEs occurred in 29% and 15% of patients, respectively. One treatment-related death, which was attributed to shock, was reported in the nivolumab plus ipilimumab arm of CheckMate 227 (Table 2).

**Table 2:** Safety summary in the pooled Japanese subpopulation with tumor PD-L1 < 1%

*n* (%)	Nivolumab plus ipilimumab with or without chemotherapy (*n* = 34)		Chemotherapy (*n* = 41)	
	Any grade	Grade 3–4	Any grade	Grade 3–4
Any TRAEs^a^	32 (94)	14 (41)	38 (93)	18 (44)
TRAEs occurring in ≥ 10% of patients in either arm in the pooled subpopulation^a^				
Malaise	10 (29)	0	10 (24)	0
Rash maculo-papular	10 (29)	2 (6)	2 (5)	0
Decreased appetite	7 (21)	0	15 (37)	3 (7)
Fatigue	7 (21)	1 (3)	3 (7)	0
Constipation	6 (18)	0	22 (54)	1 (2)
Hyperthyroidism	6 (18)	0	0	0
Pyrexia	6 (18)	1 (3)	0	0
Increased alanine aminotransferase	5 (15)	0	6 (15)	0
Rash	5 (15)	0	1 (2)	0
Alopecia	4 (12)	0	5 (12)	0
Anemia	4 (12)	1 (3)	14 (34)	6 (15)
Increased aspartate aminotransferase	4 (12)	0	4 (10)	0
Diarrhea	4 (12)	0	5 (12)	1 (2)
Pruritus	4 (12)	0	0	0
Hiccups	3 (9)	0	13 (32)	0
Nausea	3 (9)	0	21 (51)	1 (2)
Neutropenia	1 (3)	1 (3)	6 (15)	4 (10)
Decreased neutrophil count	1 (3)	1 (3)	12 (29)	6 (15)
Decreased platelet count	0	0	13 (32)	3 (7)
Decreased white blood cell count	0	0	5 (12)	3 (7)
Serious TRAEs^a^	13 (38)	10 (29)	10 (24)	6 (15)
TRAEs leading to discontinuation of any component of the study drug^a^	8 (24)	4 (12)	11 (27)	5 (12)
IMAEs^b^	23 (68)	7 (21)	1 (2)	0
Treatment-related deaths	1^c^ (3)		0	

^a^TRAEs include events reported between the first dose and 30 days after the last dose of study therapy. ^b^Included events reported between the first dose and 100 days after the last dose of study therapy. ^c^Included 1 patient from CheckMate 227 who died due to shock

*IMAE* immune-mediated adverse event; *PD-L1* programmed death ligand 1; *TRAE* treatment-related adverse event

## Discussion

To our knowledge, this is the first pooled analysis providing long-term follow-up data on clinical outcomes with a first-line dual immunotherapy-based regimen in Japanese patients with metastatic NSCLC and tumor PD-L1 < 1%. Results from this analysis showed long-term, durable efficacy benefit with nivolumab plus ipilimumab with or without chemotherapy versus chemotherapy alone, with 38% versus 16% of patients alive at 5 years, and a median DOR of 16.6 versus 3.0 months with 18% versus 0% of responders experiencing ongoing tumor response at 5 years. No new safety signals were identified. Results were consistent with those reported for the pooled global population [25].

Results from this pooled analysis were also consistent with the efficacy data on the Japanese subpopulation of CheckMate 227, with 5-year OS rates of 36% versus 19% observed with nivolumab plus ipilimumab versus chemotherapy in Japanese patients with tumor PD-L1 < 1% [29]. Additionally, efficacy outcomes from this analysis were consistent with the long-term efficacy data from patients with tumor PD-L1 < 1% in the global CheckMate 227 and CheckMate 9LA studies, with 6-year OS rates of 16% versus 5% observed with nivolumab plus ipilimumab versus chemotherapy in CheckMate 227 and 6-year OS rates of 20% versus 7% observed with nivolumab plus ipilimumab with chemotherapy versus chemotherapy in CheckMate 9LA [1, 2]. In this analysis, the Kaplan–Meier curves for OS in the pooled nivolumab plus ipilimumab with or without chemotherapy versus chemotherapy arms remained separated, with the curves plateauing over time, suggestive of long-term, durable survival benefit with nivolumab plus ipilimumab with or without chemotherapy in Japanese patients with metastatic NSCLC and tumor PD-L1 < 1%. Furthermore, this clinically meaningful OS benefit with nivolumab plus ipilimumab with or without chemotherapy was maintained despite the high rates of subsequent immunotherapy (71%) in the chemotherapy arm.

Furthermore, the long-term OS outcomes in this pooled analysis were similar to those observed among patients with tumor PD-L1 ≥ 1% in the global population treated with nivolumab plus ipilimumab versus chemotherapy in CheckMate 227 (6-year OS rates: 22% vs. 13%) and those treated with nivolumab plus ipilimumab with chemotherapy versus chemotherapy in CheckMate 9LA (6-year OS rates: 15% vs. 10%) [1, 2]. Long-term OS benefit with nivolumab plus ipilimumab with or without chemotherapy versus chemotherapy in this pooled analysis of Japanese patients with tumor PD-L1 < 1% was also consistent with that observed with nivolumab plus ipilimumab versus chemotherapy in Japanese patients from CheckMate 227 with tumor PD-L1 ≥ 1% (5-year OS rates: 46% vs. 34%), despite the numerically lower 5-year OS rate with the immunotherapy-based regimen in this pooled analysis (38%) compared with that in patients with tumor PD-L1 ≥ 1% (46%) [29]. Altogether, findings from this pooled analysis support consistent efficacy benefit with nivolumab plus ipilimumab-based treatments in Japanese patients with metastatic NSCLC, regardless of tumor PD-L1 expression.

A high proportion (~ 26%) of patients with stage IV, metastatic NSCLC present with brain metastases at diagnosis, representing a population with poor prognosis and considerable unmet need [30]. In this pooled analysis, only 12% of patients in the nivolumab plus ipilimumab with chemotherapy arm and 2% in chemotherapy arm had brain metastasis at baseline, reflecting the strict eligibility criteria of CheckMate 227 and 9LA studies, which only included patients with treated, asymptomatic brain metastases. Therefore, it cannot be discounted that the lower proportion of patients with brain metastasis at baseline in this analysis relative to real-world occurrence may have contributed to the favorable efficacy outcomes observed with nivolumab plus ipilimumab with chemotherapy. Generalizing this study’s findings to patients with active, symptomatic, or untreated brain metastases warrants further investigation.

Discontinuation of nivolumab plus ipilimumab with or without chemotherapy due to TRAEs did not appear to have a negative impact on efficacy and tumor response, when compared with the overall pooled population in the nivolumab plus ipilimumab with or without chemotherapy arm; however, it should be noted that such analyses are limited by immortal time bias. Patients in the nivolumab plus ipilimumab with or without chemotherapy arm tended to remain treatment-free for a longer duration prior to disease progression compared with patients in the chemotherapy arm who showed progression soon after treatment discontinuation.

No new safety signals were reported in this pooled analysis of Japanese patients, and safety outcomes were generally consistent with those reported for the pooled global population [25], with only one treatment-related death (due to shock) reported in the nivolumab plus ipilimumab with or without chemotherapy arm. This pooled analysis presents key AEs and serious side effects reported in the nivolumab plus ipilimumab with or without chemotherapy and chemotherapy arms, providing an impartial evaluation of the safety profiles of patients in both treatment arms.

Whether chemotherapy should be added to nivolumab plus ipilimumab in patients with tumor PD-L1 < 1% remains an important clinical question. Although cross-trial comparisons should be made with caution owing to differences in study designs and patient populations, the addition of a limited course of chemotherapy to nivolumab plus ipilimumab in CheckMate 9LA appeared to provide greater response benefit than nivolumab plus ipilimumab in CheckMate 227 in patients with tumor PD-L1 < 1%; 6-year ORR was 31% with nivolumab plus ipilimumab with chemotherapy and 27% with nivolumab plus ipilimumab [1, 2]. Nevertheless, long-term survival outcomes were similar for the two immunotherapy-based regimens in patients with tumor PD-L1 < 1%. In this pooled analysis, nivolumab plus ipilimumab with or without a limited course of chemotherapy demonstrated long-term survival benefit versus chemotherapy alone. However, the small sample size and exploratory nature of this post hoc analysis limited direct comparison of nivolumab plus ipilimumab with versus without chemotherapy. The addition of chemotherapy may contribute to early disease control, whereas dual immunotherapy alone may provide durable benefit in selected patients. Therefore, treatment selection should be individualized, considering disease burden, patient condition, and the need for rapid tumor control. Furthermore, no clinical trials have compared nivolumab plus ipilimumab with nivolumab plus ipilimumab with chemotherapy so far, which limits definitive conclusions.

This analysis had several limitations. The study was not statistically powered to evaluate differences between treatment arms. Furthermore, small sample sizes limit data interpretation and generalization. This analysis was also limited by differences between the CheckMate 227 and CheckMate 9LA studies, such as patient populations, study designs, treatment regimens, and nivolumab dosing, which may have introduced heterogeneity in the pooled arms.

In this pooled analysis of CheckMate 227 and CheckMate 9LA, first-line nivolumab plus ipilimumab with or without chemotherapy versus chemotherapy alone was associated with long-term, durable efficacy benefit in Japanese patients with metastatic NSCLC and tumor PD-L1 < 1%, consistent with results in the global population. These findings suggest potential clinical benefit with the use of nivolumab plus ipilimumab with or without chemotherapy as first-line treatment for Japanese patients with metastatic NSCLC and tumor PD-L1 < 1%, a population with high unmet need.

## Supplementary Information

Below is the link to the electronic supplementary material.

Supplementary Material 1

## Data Availability

Data are available on reasonable request. BMS policy on data sharing may be found at https://www.bms.com/researchers-and-partners/independent-research/data-sharing-request-process.html.
