# Supplementary material for: Nivolumab plus ipilimumab-based treatment in Japanese patients with metastatic non-small cell lung cancer and tumor PD-L1 < 1%: a pooled analysis
Source: Int J Clin Oncol. 2026 Aug 11;31(10):2179–90. doi: 10.1007/s10147-026-03116-w (PMC13615008; doi:10.1007/s10147-026-03116-w)
Supplement: Supplementary file 1 [file 10147_2026_3116_MOESM1_ESM.docx]

**SUPPLEMENTARY INFORMATION**

**Nivolumab plus ipilimumab-based treatment in Japanese patients with metastatic non-small cell lung cancer and tumor PD-L1 <1%: A pooled analysis**

Makoto Nishio^1^ · Hidetoshi Hayashi^2^ · Yasushi Goto^3^ · Satoshi Ikeda^4^ · Toshihide Yokoyama^5^ · Hiroshi Tanaka^6^ · Yuichi Tambo^7^ · Katsuyuki Hotta^8^ · Tatsuro Fukuhara^9^ · Haruko Daga^10^ · Ken Masubuchi^11^ · Takekazu Aoyama^12^ · Marina Tschaika^12^ · Nan Hu^12^ · Hideaki Mizutani^13^

^1^Thoracic Medical Oncology Department, Cancer Institute Hospital of the Japanese Foundation for Cancer Research, Tokyo, Japan

^2^Department of Medical Oncology, Kindai University, Faculty of Medicine, Osaka, Japan

^3^Department of Thoracic Oncology, National Cancer Center Hospital, Tokyo, Japan

^4^Department of Respiratory Medicine, Kanagawa Cardiovascular and Respiratory Center, Yokohama, Kanagawa, Japan

^5^Department of Respiratory Medicine, Kurashiki Central Hospital, Kurashiki, Okayama, Japan

^6^Department of Internal Medicine, Niigata Cancer Center Hospital, Chuo Ward, Niigata, Japan

^7^Department of Respiratory Medicine, Kanazawa University Hospital, Kanazawa, Ishikawa, Japan

^8^Department of Respiratory Medicine, Okayama University Hospital, Kita Ward, Okayama, Japan

^9^Tatsuro Fukuhara, Miyagi Cancer Center, Natori, Miyagi, Japan

^10^Department of Medical Oncology, Osaka City General Hospital, Chuo Ward, Osaka, Japan

^11^Division of Respiratory Medicine, Gunma Prefectural Cancer Center, Ota, Gunma, Japan

^12^Bristol Myers Squibb Company, Princeton, New Jersey, USA

^13^Department of Thoracic Oncology, Saitama Cancer Center, Kitaadachi District, Saitama, Japan

**Corresponding author:**

Makoto Nishio, MD, PhD

Cancer Institute Hospital of the Japanese Foundation for Cancer Research

3-8-31, Ariake, Koto, Tokyo, 135-8550, Japan.

Email: mnishio@jfcr.or.jp; Telephone number: +81-3-3520-0111; Fax number: +81-3-3520-0141

**SUPPLEMENTARY TABLES**

| **Supplementary Table 1.** Treatment and exposure summary in the pooled Japanese subpopulation with tumor PD-L1 <1%. | | |
| --- | --- | --- |
|  | **Pooled Japanese subpopulation (*n* = 75)** | |
| Duration of treatment, median (range), months | 3.5 (0.0–24.3) | |
|  | ***n*** | **Number of doses,  median (range)** |
| Nivolumab | 34 | 8 (1–53) |
| Ipilimumab | 34 | 4 (1–18) |
| Gemcitabine | 5 | 6 (1–8) |
| Cisplatin | 34 | 3 (1–4) |
| Carboplatin | 18 | 4 (1–4) |
| Pemetrexed | 40 | 4 (1–34) |
| Paclitaxel | 5 | 2 (1–4) |
| *PD-L1* programmed death ligand 1. | | |

| **Supplementary Table 2.** Subsequent therapy in the pooled Japanese subpopulation with tumor PD-L1 <1%. | | |
| --- | --- | --- |
| ***n* (%)** | **Nivolumab plus ipilimumab with or without chemotherapy (*n* = 34)** | **Chemotherapy (*n* = 41)** |
| Any subsequent therapy | 28 (82) | 38 (93) |
| Subsequent radiotherapy | 15 (44) | 12 (29) |
| Subsequent surgery | 3 (9) | 0 (0) |
| Subsequent systemic therapy | 27 (79) | 34 (83) |
| Immunotherapy | 7 (21) | 29 (71) |
| Anti-PD-1 | 4 (12) | 24 (59) |
| Nivolumab | 3 (9) | 19 (46) |
| Pembrolizumab | 1 (3) | 5 (12) |
| Anti-PD-L1 | 5 (15) | 5 (12) |
| Atezolizumab | 4 (12) | 5 (12) |
| Durvalumab | 1 (3) | 0 |
| Anti-CTLA-4 | 1 (3) | 0 |
| Ipilimumab | 1 (3) | 0 |
| Targeted therapy | 0 | 3 (7) |
| Chemotherapy^a^ | 27 (79) | 22 (54) |
| Pemetrexed | 18 (53) | 2 (5) |
| Carboplatin | 14 (41) | 4 (10) |
| Docetaxel | 13 (38) | 17 (42) |
| Cisplatin | 11 (32) | 2 (5) |
| Paclitaxel | 11 (32) | 5 (12) |
| Gemcitabine | 4 (12) | 3 (7) |
| Gimer/Otera/Tegfur | 4 (12) | 4 (10) |
| Gimeracil; Oteracil potassium; Tegafur | 4 (12) | 2 (5) |
| Patients may have received more than 1 type of subsequent therapy.  ^a^Received by >10% of patients in either treatment arm.  *CTLA-4* cytotoxic T-lymphocyte antigen 4; *PD-1* programmed death 1; *PD-L1* programmed death ligand 1. | | |

| **Supplementary Table 3.** ORR, BOR, and DOR per BICR in the pooled Japanese subpopulation with tumor PD-L1 <1%. | | | | |
| --- | --- | --- | --- | --- |
|  | **Overall** | | **Discontinued due to TRAEs^a^** | |
|  | **Nivolumab plus ipilimumab with or without chemotherapy (*n* = 34)** | **Chemotherapy  (*n* = 41)** | **Nivolumab plus ipilimumab with or without chemotherapy (*n* = 12)** | **Chemotherapy  (*n* = 11)** |
| ORR, *n* (%) | 12 (35) | 13 (32) | 5 (42) | 5 (45) |
| (95% CI) | (20–54) | (18–48) | (15–72) | (17–77) |
| BOR, *n* (%) | | | | |
| CR | 3 (9) | 0 | 1 (8) | 0 |
| PR | 9 (26) | 13 (32) | 4 (33) | 5 (45) |
| SD | 13 (38) | 24 (59) | 6 (50) | 6 (55) |
| PD | 7 (21) | 3 (7) | 1 (8) | 0 |
| Unable to determine | 2 (6) | 1 (2) | 0 | 0 |
| Median DOR, mo | 16.6 | 3.0 | 33.8 | 5.3 |
| (95% CI) | (7.2–51.1) | (2.8–5.3) | (8.3–NR) | (2.7–NR) |
| ^a^Post hoc analysis. Patients discontinued all components of nivolumab plus ipilimumab with or without chemotherapy due to TRAEs.  *BICR* blinded independent central review; *BOR* best overall response; *CI* confidence interval; *CR* complete response; *DOR* duration of response; *NR* not reached; *ORR* objective response rate; *PD* progressive disease; *PR* partial response; *PD-L1* programmed death ligand 1; *SD* stable disease; *TRAE* treatment-related adverse event. | | | | |

**FIGURES**

**Supplementary Fig. 1** Patient disposition in the pooled Japanese subpopulation with tumor PD-L1 <1%. Treatment continued until disease progression, unacceptable toxicity, or for 2 years for immunotherapy. ^a^NCT02477826. ^b^NCT03215706. ^c^Determined by the PD-L1 IHC 28–8 pharmDx assay (Dako). ^d^Nivolumab (360 mg Q3W). ^e^NSQ: pemetrexed + cisplatin or carboplatin, Q3W for ≤ 4 cycles, with optional pemetrexed maintenance following chemo or nivolumab + pemetrexed maintenance following nivolumab + chemotherapy; SQ: gemcitabine + cisplatin, or gemcitabine + carboplatin, Q3W for ≤ 4 cycles. ^f^Nivolumab (3 mg/kg Q2W) + ipilimumab (1 mg/kg Q6W). ^g^Nivolumab (360 mg Q3W) + ipilimumab (1 mg/kg Q6W) + chemotherapy (Q3W for 2 cycles). ^h^NSQ: pemetrexed + cisplatin or carboplatin; SQ: paclitaxel + carboplatin. ^i^Chemotherapy (Q3W for 4 cycles); optional pemetrexed maintenance (500 mg/m^2^) was permitted in patients with NSQ histology. *Chemo* chemotherapy*; IHC* immunohistochemistry; *IPI* ipilimumab; *ITT* intent to treat; *NIVO* nivolumab; *NSQ* non-squamous; *PD-L1* programmed death ligand 1; *Q3W* every 3 weeks; *R* randomization; *SQ* squamous.

**Supplementary Fig. 2** Treatment status, response, and key baseline characteristics of individual patients in the nivolumab plus ipilimumab with or without chemotherapy arm in the pooled Japanese subpopulation with tumor PD-L1 < 1%, who did not receive any subsequent therapy. *CR* complete response; *DBL* database lock; *ECOG PS* Eastern Cooperative Oncology Group performance status; *PD* progressive disease; *PD-L1* programmed death ligand 1; *PR* partial response; *SD* stable disease, *TRAE* treatment-related adverse event.
